# A Lower Global Lung Ultrasound Score Is Associated with Higher Likelihood of Successful Extubation in Invasively Ventilated COVID-19 Patients

**DOI:** 10.4269/ajtmh.21-0545

**Published:** 2021-10-18

**Authors:** Charalampos Pierrakos, Arthur Lieveld, Luigi Pisani, Marry R. Smit, Micah Heldeweg, Laura A. Hagens, Jasper Smit, Mark Haaksma, Lars Veldhuis, Robin Walburgh Schmidt, Giacomo Errico, Valentina Marinelli, Rachid Attou, Cristina E. David, Claudio Zimatore, Francesco Murgolo, Salvatore Grasso, Lucia Mirabella, Gilda Cinnella, David De Bels, Marcus J. Schultz, Pieter-Roel Tuinman, Lieuwe D. Bos

**Affiliations:** ^1^ Department of Intensive Care and Laboratory of Experimental Intensive Care and Anesthesiology, Amsterdam University Medical Centers—AMC, Amsterdam, The Netherlands;; ^2^ Department of Intensive Care, Brugmann University Hospital, Université Libre de Bruxelles, Belgium;; ^3^ Department of Intensive Care and Acute Internal Medicine, Amsterdam University Medical Centers—VUMC, Amsterdam, The Netherlands;; ^4^ Department of Anesthesia and Intensive Care, Miulli Regional Hospital, Acquaviva delle Fonti, Italy;; ^5^ Mahidol–Oxford Tropical Medicine Research Unit, Mahidol University, Bangkok, Thailand;; ^6^ Department of Anesthesia and Intensive Care, Foggia University Hospital, University of Foggia, Italy;; ^7^ Intensive Care Unit, Department of Emergency and Organ Transplantation, University of Bari, Bari, Italy;; ^8^ Nuffield Department of Medicine, University of Oxford, United Kingdom;; ^9^ ALIFE, Amsterdam Leiden IC Focussed Echograpy, Amsterdam, The Netherlands

## Abstract

Lung ultrasound (LUS) can be used to assess loss of aeration, which is associated with outcome in patients with coronavirus disease 2019 (COVID-19) presenting to the emergency department. We hypothesized that LUS scores are associated with outcome in critically ill COVID-19 patients receiving invasive ventilation. This retrospective international multicenter study evaluated patients with COVID-19–related acute respiratory distress syndrome (ARDS) with at least one LUS study within 5 days after invasive mechanical ventilation initiation. The global LUS score was calculated by summing the 12 regional scores (range 0–36). Pleural line abnormalities and subpleural consolidations were also scored. The outcomes were successful liberation from the ventilator and intensive care mortality within 28 days, analyzed with multistate, competing risk proportional hazard models. One hundred thirty-seven patients with COVID-19–related ARDS were included in our study. The global LUS score was associated with successful liberation from mechanical ventilation (hazard ratio [HR]: 0.91 95% confidence interval [CI] 0.87–0.96; *P* = 0.0007) independently of the ARDS severity, but not with 28 days mortality (HR: 1.03; 95% CI 0.97–1.08; *P* = 0.36). Subpleural consolidation and pleural line abnormalities did not add to the prognostic value of the global LUS score. Examinations within 24 hours of intubation showed no prognostic value. To conclude, a lower global LUS score 24 hours after invasive ventilation initiation is associated with increased probability of liberation from the mechanical ventilator COVID–19 ARDS patients, independently of the ARDS severity.

## INTRODUCTION

Coronavirus disease 2019 (COVID-19) is responsible for hundreds of thousands of deaths worldwide, and this number is still rapidly increasing.^1^ Respiratory failure is the most common and severe complication of COVID-19, and bilateral and multilobar infiltrates can progress rapidly over the first few days of illness.^2^ Approximately 5% of hospitalized patients require admission to an intensive care unit (ICU), mainly for invasive mechanical ventilation.^3^ There is a high variability in the reported mortality across invasively ventilated COVID-19 patients.^4^^–^^6^ The severity of loss of aeration, typically assessed by chest computed tomography (CT) scan, has been related to outcomes in hospitalized COVID-19 patients,^7^^–^^9^ but CT-scan capacity is limited and may not be available in resource-limited settings.

Lung ultrasound is a bedside, radiation-free, low-cost diagnostic imaging tool that can be used for assessing lung aeration and parenchymal abnormalities.^10^ The global lung ultrasound score (LUS) quantifies lung aeration by translating LUS patterns into a numerical score across 12 lung regions and summing the results.^11^^,^^12^ Previous studies have shown a correlation between LUS and severity of acute respiratory distress syndrome (ARDS),^13^ and with mortality in invasively ventilated critically ill patients.^14^ LUS has previously been performed in a general population of COVID-19 patients outside the hospital,^15^ presenting to the emergency department (ED)^16^^–^^18^ and on the general wards^19^^–^^26^ and has been found to be related to adverse outcomes including the need of invasive ventilation. Nevertheless, the role of LUS in evaluating the severity of patients after initiation of invasive ventilation is much less certain.^27^ Evaluating lung disease severity with LUS may be important for ICU resource planning, especially in settings where resources are restricted, as well as for personalized ventilatory approach.^28^

The aim of this study is to assess the association between global LUS score and outcome, specifically defined as liberation from the ventilator and survival in critically ill COVID-19 patients under invasive ventilation. We hypothesized the global LUS score to have prognostic value in invasively ventilated COVID-19 patients independently of ARDS severity.

## METHODS

### Design.

This is an international multicenter cohort study. We retrospectively reviewed patients with reverse transcriptase polymerase chain reaction confirmed COVID-19 under invasive ventilation in ICUs of four hospitals in three countries: the Brugmann University Hospital Brussels (Brussels, Belgium), the Miulli Regional Hospital (Acquaviva delle Fonti, Italy), and the Amsterdam University Medical Centers, locations AMC and VUMC, between February 2020 and December 31, 2020. LUS studies were performed as part of routine practice and were executed by experienced ultrasonographers (*N* = 10) who performed at least 50 systematic lung ultrasound exams before.

### Ethics.

Ethical approval for this study was provided by the ethical committees of each hospital (Brugmann University Hospital No. CE 2020/136; Azienda Ospedaliero-Universitaria Policlinico di Bari 0030638/22/04/2020; Amsterdam UMC location AMC W18_311; Amsterdam UMC location VUMC 2020.011).

### Patients.

Patients were included if they received an LUS within first 5 days after invasive ventilation initiation but before extubation. Patients who received a LUS while supported with extracorporeal membrane oxygenation were excluded. Only the first available examination of LUS was used for the analysis.

### Data collected.

The following data were extracted from chart: demographic characteristics, APACHE II and SOFA score on admission, vital signs, and ventilator and blood gas parameters on the day of LUS examination.

### The protocol for LUS.

Lung ultrasound examination was performed with the available equipment at the COVID ICUs of the participating hospitals. The following machines were available: MyLab Five ultrasound machine with a convex probe (Esaote Spa, Genova, Italy), a Vivid S5 with curvilinear probe (General Electric Healthcare, Chicago, IL), a LOGIQ E with a linear probe (GE Healthcare, Milwaukee, WI), and a Sonosite Edge II (Fujifilm Sonosite, Bothell, WA).

**Figure 1.**
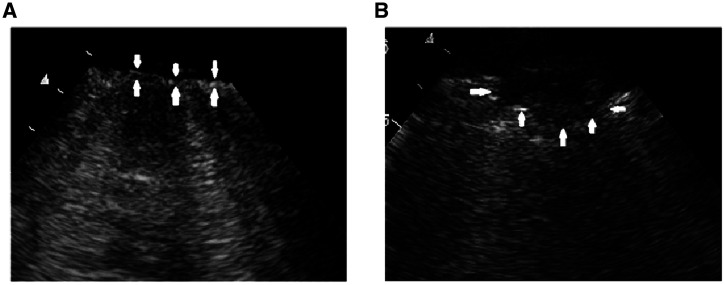
Pleural line abnormalities (**A**) and subpleural consolidation (**B**) identified with lung ultrasound in patient with COVID-19 infection.

The 12-region technique was used in all examinations in which ultrasound was performed on six areas on each side of the chest: two ventral regions, two lateral regions, and two posterolateral regions.^29^ The aeration pattern observed in each region was scored from 0 to 3 according to the LUS aeration score as follows: 0 = A pattern with ≤2 B lines; 1 = >2 separated B lines that cover ≤50% of the pleural line; 2 = B lines that cover >50% of the pleural line; or 3 = lung consolidation. In theory, the global LUS score can range from 0 (normal aeration in all regions) to 36 (severe abnormal aeration in all regions).^30^ Presence of subpleural consolidations and abnormal pleural line was also assessed offline in each field using saved ultrasound clips. Thickening and fragmentation of the pleural line and the finding of subpleural consolidations do not currently contribute to the LUS aeration score but are often found in patients with COVID-19 infection. Therefore, we assessed the number of fields with pleural line abnormalities and the number of fields with subpleural consolidations in a separate analysis. Pleural line abnormalities were defined as any deviation from the normally appearing thin, smooth and continuous hyperechoic line (Figure 1).^29^ Subpleural consolidations were defined as one or more echo poor regions juxtaposed to the pleural line, which were not large enough to be scored as a tissue-like pattern or “lobar consolidation” in the lung aeration score (Figure 1).^30^

### Outcomes.

The primary outcomes were the risks for successful liberation from invasive ventilation and intensive care mortality up to 28 days.

### Statistical analysis.

No formal power analysis was performed for this exploratory analysis. Rather, all patients that fulfilled the inclusion and exclusion criteria were included. Demographic, clinical, and outcome variables were presented as percentages for categorical variables and as medians with interquartile ranges (IQR) for continuous variables.

The association of LUS with outcomes was analyzed with multistate, competing risk proportional hazard models as described in the *survival* package via the *compete* function in *R*. Risks were estimated for successful extubation and mortality and compared with persistent intubation (reference category). Follow-up was censored after 28 days. Two sensitivity analysis were performed for the following predefined subgroups: 1) severity of ARDS according to PaO_2_/FiO_2_ based on cutoffs described in the Berlin definition^31^ and 2) days of invasive ventilation before LUS examination (exam on day 0, day 1, day 2–3, day 4–5).

Receiver operating characteristic (ROC) curve analysis was used to derive the prognostic discriminatory performance of global LUS in determining successful liberation from invasive ventilation and mortality at day 28. On the basis of the calculated area under the ROC curve (AUROCC), the prognostic accuracy was interpreted as follows: excellent between 0.9 and 1, good between 0.8 and 0.89, fair between 0.7 and 0.79, poor between 0.6 and 0.69, and very poor between 0.5 and 0.59.^32^ AUROCCs were compared using the DeLong test. Two cutoffs were defined: one with a high sensitivity of > 80% for poor outcome (composite of persistent mechanical ventilation at day 28 or mortality; selecting for a good negative predictive value) and one with a high specificity of above 80% for poor outcome (selecting for a good positive predictive value). The analysis was repeated using the three categories resulting from these cutoffs. All analyses were performed in R through the R-studio interface (www.r-project.org, R version 3.3.1). A *P* value < 0.05 was considered significant.

## RESULTS

### Patients.

The study included 137 patients. Patient characteristics are presented in Table 1. At day 28, 53 patients (38%) were successfully extubated, 64 patients (47%) had died, and 20 patients (15%) were still receiving invasive mechanical ventilation. Compared with the patients who failed to be extubated within 28 days, patients who were successfully extubated had a higher PaO_2_/FiO_2_ of 148 mm Hg (IQR: 115–173) versus 113 mm Hg (IQR: 98–153, *P* = 0.013) and a lower global LUS score of 18 (IQR: 15–23) versus 21 points (IQR: 18–24, *P* = 0.005).

**Table 1:** Characteristics of patients with COVID-19 disease examined with lung ultrasound

	Successful liberation of mechanical ventilation and alive at 28 days	Still intubated or deceased at 28 days	*P* value
Number of patients	53	84	
Day of LUS	1 (0–2)	1 (0–2)	0.71
Demographics			
Age, years	61 (54–68)	71 (62–76)	< 0.01
Sex, female	32 (61)	46 (54)	0.59
APACHE II	13 (12–17)	14 (12–22)	0.21
SOFA score	7 (5–8)	8 (5-10)	0.06
Global LUS score	18 (15–23)	21 (18–24)	< 0.01
Subpleural consolidations, fields per patient	3 (1–5)	5 (2–6)	0.01
Pleural line abnormalities, fields per patient	3 (1–5)	3 (1–5)	0.91
Biology			
D-dimers, ng/mL	1,440 (873–3,875)	2,326 (1720–4,063)	0.03
CRP, µg/mL	77 (40–159)	108 (21–172)	0.81
Ventilation parameters			
Ventilation mode			
Volume controlled	20 (38)	36 (42)	0.59
Pressure controlled	17 (32)	30 (36)	0.71
Pressure support	16 (30)	18 (22)	0.31
Tidal volume, ml	425 (378–484)	418 (390–460)	0.71
Tidal volume, ml/kg PBW	6.3 (5.8–6.9)	6.3 (5.6**–**6.5)	0.75
PEEP set, cm H_2_O	10 (8–12)	10 (8–12)	0.81
Pplat, cm H_2_O	22 (19–26)	24 (20–27)	0.21
Driving Pressure	12 (11–17)	14 (10–17)	0.55
Static Compliance	35 (26–44)	31 (24–44)	0.55
SpO_2_, %	94 (93–96)	95 (94–97)	0.83
FiO_2_	55 (50–70)	64 (55–80)	< 0.01
PaO_2_/FiO_2_	148 (115–173)	113 (98–153)	0.01
Complications/procedures			
VAP	11 (21)	59 (71)	< 0.01
ECMO	0 (0)	4 (5)	0.15
Tracheostomy	5 (9)	19 (22)	0.06
Death (28 days)	0 (0)	64 (76)	

Data are presented as mean (±SD) median (interquartile range) or number (%). APACHE = Acute Physiology and Chronic Health Evaluation; ECMO = extracorporeal membrane oxygenation; FiO_2_ = fraction of inspired oxygen; ICU = intensive care unit; LUS = lung ultrasound; PaO_2_ = arterial oxygen tension; PBW = predicted body weight; PEEP = positive end–expiratory pressure; Pplat = plateau pressure; SOFA = Sequential Organ Failure Assessment; SpO_2_ = peripheral pulse oxymetric saturation; VAP = ventilator associated pneumonia.

### Association between global LUS score and outcome.

**Figure 2.**
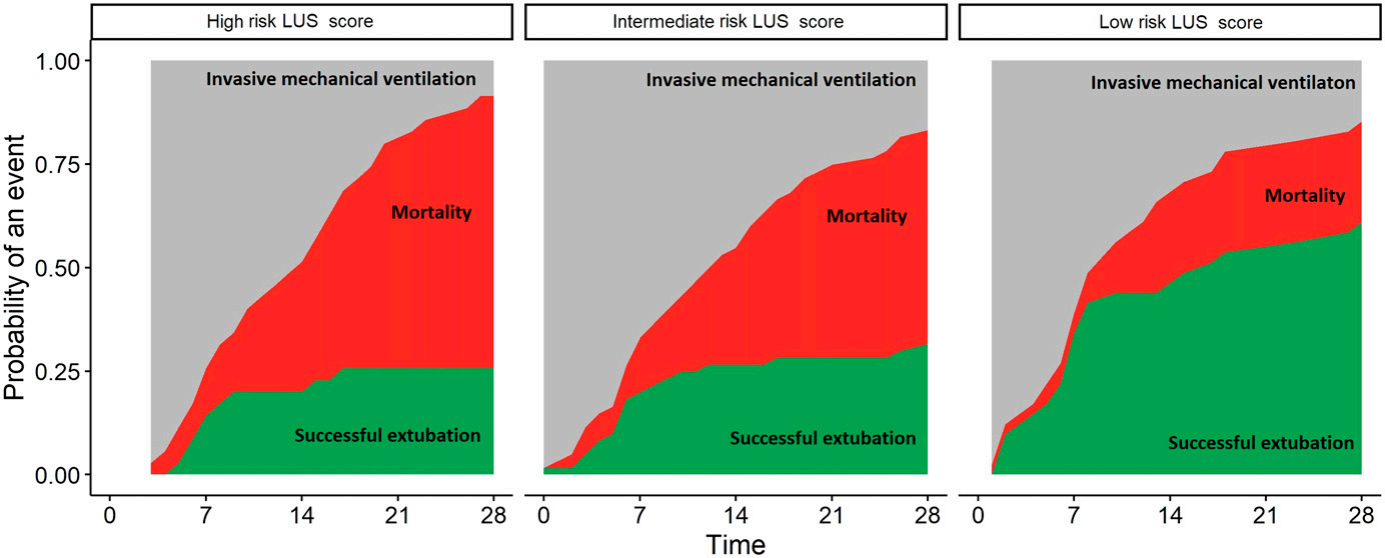
Three categories of global lung ultrasound (LUS) score and cumulative incidence of outcomes. x-axis: days since intubations; y-axis: probability of an event (extubation or death) in the population. The three facets show the risk for patients with a high risk global LUS score (left), intermediate risk (middle) and low risk global LUS score (right). Red area show the patients who died. Green area shows the patients who were successfully extubated. This figure appears in color at www.ajtmh.org.

The global LUS score was associated with successful liberation from invasive ventilation (hazard ratio [HR]: 0.91; 95% confidence interval [CI]: 0.87–0.96; *P* = 0.0007) but not with 28 days mortality (HR: 1.03; 95% CI: 0.97–1.08; *P* = 0.36) in competing risk analysis. However, the prognostic capacity of the global LUS sore for successful liberation from the ventilator at day 28 and mortality was poor (AUROCC of 0.65; 95% CI 0.54–0.74; AUROCC of 0.63; 95% CI 0.53–0.72, respectively). The optimal cutoff for > 80% sensitivity for the combined endpoint of persistent mechanical ventilation or death at day 28 was 17, whereas it was 24 for > 80% specificity (Table 2). The corresponding HRs for the probability of being liberated from the ventilator compared with a score of 17 or below (low risk group, *N* = 41), were 0.47 (95% CI: 0.26–0.85; *P* = 0.01) for the patients with LUS score 18 to 24 (intermediate risk group, *N* = 71) and 0.37 (95% CI: 0.17–0.79; *P* = 0.01) for the patients with LUS above 24 (high risk group, *N* = 25; Figure 2). Only patients with a global LUS score of 24 and above had an increased probability of death (HR: 2.3; 95% CI: 1.08–4.8; *P* = 0.03) compared with a score of 17 or below. Adding subpleural consolidations or pleural line abnormalities did not improve the prognostic capacity for successful extubation or mortality at day 28 (Table 2).

**Table 2:** Prognostic values of global lung ultrasound (LUS) score alone and with the addition of number of areas with subpleural consolidations or pleural line abnormalities

Outcome	Score	AUROCC (95% CI)	Cutoff		Poor outcome	Good outcome
Successful liberation from mechanical ventilation while alive at day 28	Global LUS score	0.65 (0.54–0.74)	17	≥ 17	68	28
				< 17	16	25
					Sensitivity: 81	Specificity: 47
			24	≥ 24	26	9
				< 24	58	44
					Sensitivity: 31	Specificity: 83
	+ Subpleural consolidations	0.65 (0.55–0.75)	19	≥ 19	62	29
				< 19	12	20
					Sensitivity: 84	Specificity: 41
			28	≥ 28	25	7
				< 28	49	42
					Sensitivity: 32	Specificity: 86
	+ Pleural line abnormalities	0.61 (0.51–0.71)	18	≥ 18	61	32
				< 18	13	17
					Sensitivity: 82	Specificity: 35
			28	≥ 28	13	17
				< 28	53	40
					Sensitivity: 28	Specificity: 82
Mortality at day 28	Global LUS score	0.63 (0.53–0.72)	17	≥ 17	54	42
				< 17	10	31
					Sensitivity: 84	Specificity: 37
			24	≥ 24	23	12
				< 24	41	61
					Sensitivity: 35	Specificity: 83
	+ Subpleural consolidations	0.61 (0.51–0.71)	19	≥ 19	46	45
				< 19	10	22
					Sensitivity: 82	Specificity: 32
			28	≥ 28	19	13
				< 28	37	54
					Sensitivity: 33	Specificity: 81
	+ Pleural line abnormalities	0.58 (0.47–0.68)	18	≥ 18	46	47
				< 18	10	20
					Sensitivity: 82	Specificity: 30
			28	≥ 28	19	11
				< 28	37	56
					Sensitivity: 33	Specificity: 83

Prognostic accuracy stratified for successful liberation from invasive ventilation and mortality at day 28. Areas under the ROC curve (AUROCC) with optimal cutoff and corresponding sensitivity and specificity are shown for the global LUS score alone, and for the score in combination with subpleural consolidations and pleural line abnormalities. The *P*-value indicates the difference between the global LUS score and the score combined with either additional finding.

### Subgroup analyses.

**Figure 3.**
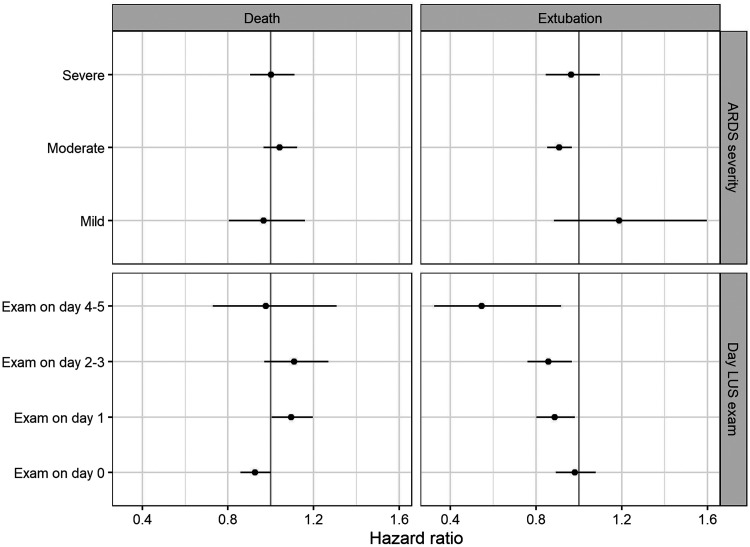
Forest plot of global lung ultrasound (LUS) score association with probability of successful liberation of invasive ventilation and death at 28 days according to acute respiratory distress syndrome (ARDS) severity and the day of examination after start invasive ventilation. x-axis: hazard ratio for increase of global LUS score for mortality (left) and extubation (right) based on competing risk analysis. The dots provide the point estimate and the lines the 95% confidence interval for estimated associations, stratified for predefined subgroups.

When patients were classified according to ARDS severity, 11 patients (9%) had mild, 85 patients (68%) had moderate, and 29 (23%) had severe ARDS. For 12 patients PaO_2_/FiO_2_ was missing. The global LUS score was associated with the probability of being liberated from the ventilator while alive at 28 days after intubation independently of ARDS severity (Figure 3). There was no evidence for variation of global LUS association with outcome according to the categories of P_a_O_2_/FiO_2_ (no interaction; *P* = 0.49).

The majority of the patients were examined within 24 hours after invasive ventilation initiation (61 patients, 44%), whereas 39 patients (28%) were examined between 24 and 48 hours, 24 patients (17%) between 48 and 96 hours, and 13 patients (9%) between 96 and 120 hours after start of invasive ventilation. There was evidence variation of global LUS score according to the time of examination (interaction term, *P* = 0.036). When the examination was performed within 24 hours after intubation, the global LUS score was not associated with the probability of successful extubation in the first 28 days (Figure 3).

## DISCUSSION

The results of this study can be summarized as follows: 1) an increasing global LUS score indicative of parenchymal damage and loss of aeration is associated with a lower likelihood of mechanical ventilation liberation during the first 28 days of invasive mechanical ventilation but not with mortality, 2) this association was independent from ARDS severity but not from timing of examination, and 3) additional LUS findings such as subpleural consolidations and pleural line abnormalities do not significantly improve the prognostic value.

LUS is attractive method for evaluation the severity of COVID-19 patients because ultrasound machines are widely available, and thus the technique can be used even in resource-limited settings. Furthermore, LUS examination at the bedside can potentially decrease or eliminate the need for transport to the radiology department, which is particularly helpful in invasively ventilated patients. Increasing global LUS scores were associated with a higher probability of requiring invasive ventilation for at least 28 days.

Severity assessment of severe COVID-19 early after invasive ventilation initiation is challenging as ventilator management can moderate the prognostic value of easily derived parameters such as the P_a_O_2_/FiO_2._^33^ Simultaneously, the compliance of the respiratory system is low in most of these patients and has little prognostic value.^34^ In our study, patients who successfully extubated had a similarly low respiratory system compliance compared with patients who were not successfully extubated. The loss of aeration estimation with LUS was associated with successful extubation independent of the P_a_O_2_/FiO_2_ categories, which are used in clinical practice for ARDS severity assessment. Hence, on the basis of these results, we think LUS can be used as an additional tool to clinical and laboratory parameters for the severity appreciation on invasively ventilated patients with COVID-19 ARDS.

We did not confirm the results of a previous study in non–COVID-19 ARDS patients that showed that a global LUS score of 16.5 was predictive for mortality.^35^ Because rapid extubation is predicted by less extensive parenchymal involvement, reflective of a lower degree of lung injury, we may speculate that mortality is mainly driven by the occurrence of ICU-acquired complications such as pneumonia, pulmonary embolism, ICU-acquired weakness, and the ability to endure prolonged duration of mechanical ventilation that is frequently needed for COVID-19–related ARDS. This finding is in line with a previous study that showed no association between decreased volume of well-aerated lung tissue as assessed by chest computed tomography and 30-day mortality in critically ill patients with COVID-19 ARDS.^36^ Of note, that study also showed that global LUS score was a better predictor of outcome than the CT severity score.^36^ In non–COVID-19 ARDS, the relationship between mortality rates and the amount of not aerated areas in invasively ventilated patients is not clear either.^37^^–^^40^ Even though the limited duration of follow-up of 1 month may have obscured associations with longer term mortality,^41^ we conclude that the extent of parenchymal involvement is a poor predictor of outcome when applied to a cohort of critically ill patients requiring invasive mechanical ventilation.

We aimed to facilitate bedside estimation for the risk of adverse outcomes by identifying a cutoff for the global LUS score that could predict mortality or liberation of invasive ventilation in COVID-19 related ARDS patients. In contrast to previous studies that evaluated patients who did not receive invasive mechanical ventilation,^17^^,^^19^^,^^23^^–^^25^ we were unable to provide a single LUS value that was highly predictive of outcome. However, liberation of mechanical ventilation at 28 days was much more likely in patients with a global LUS lower than 17, whereas this was unlikely in patients with a global LUS score greater than 24. Importantly, these cutoffs are not intended to replace continuous value and are arbitrary. We prespecified a sensitivity and specificity of 80% for successful extubation, but one could argue that higher certainties are needed for clinical decision-making.

Global LUS scores obtained from exams that were performed on the first day of invasive ventilation showed to have less prognostic value compared with LUS exams that were performed after the first day. We postulate that the association between global LUS scores and outcomes are influenced by the response to invasive mechanical ventilation and/or corticosteroid treatment. In our cohort, patients examined on the first day after invasive ventilation initiation had a median LUS score of 22. This score is consistent with the results of previous study in which patients with COVID-19 in the ED who required invasive ventilation had a median LUS score of 22.^19^ Assessment of lung reaeration as response by computed tomography in COVID-19 patients has shown conflicting results.^42^^,^^43^ Assessment of the influence of ventilator management on association between global LUS scores and outcomes should be a topic for future studies.

Subpleural consolidations and pleural line abnormalities are commonly used to distinguish ARDS from cardiogenic pulmonary edema^44^ and are also frequently reported in patients with COVID-19–related ARDS.^45^^–^^48^ In theory, both of these findings can be related to the severity of COVID-19 disease. Additionally, the presence of subpleural consolidations might also indicate a pulmonary embolism.^49^ In terms of prognostication, both subpleural consolidations and pleural line abnormalities were found to be associated with the prognosis in COVID-19 patients outside^15^ and inside the hospital.^19^ We found more regions with subpleural consolidations in patients with a poor outcome but not more pleural line abnormalities. Nevertheless, the extent of subpleural consolidations or pleural line abnormalities were not quantitatively related to liberation of ventilation or mortality in the subset of COVID-19 patients who require intubation and mechanical ventilation. Because the global LUS score was associated with the extent of subpleural consolidations, we reason that subpleural consolidations are more related to the degree of lung aeration loss rather than to a distinct predictor of mortality or liberation of invasive ventilation.

The main strength of this study is that the global LUS score was assessed by an identical and systematic method by multiple investigators in patients who were treated in four centers in three countries. Moreover, we included only COVID-19 patients with severe respiratory failure undergoing invasive ventilation, a homogeneous group of patients that has been underrepresented in COVID-19 LUS cohorts. We accounted for competing risks and were able to distill an association with liberation of ventilation when accounting for the occurrence of mortality during the first 28 days of invasive ventilation. This study also has limitations. First, this is a retrospective study, and the indication and time point for a lung ultrasound exam was not prescribed in a protocol. Lung ultrasound exams were done as part of routine clinical practice and all performed within 5 days after start of invasive ventilation. Second, we could not assess the prognostic value of the changes in the LUS score over time as LUS exams were not performed repeatedly in the participating centers. Dynamic changes in LUS scores should be studied further as monitoring tool for reaeration of lung tissue.^50^ Additional studies should focus on LUS because it is an excellent technique to use in a resource-limited setting as alternative for chest radiography or chest CT.^51^

## CONCLUSIONS

The global LUS score is associated with successful liberation from invasive ventilation but not with mortality during the first 4 weeks of invasive ventilation. In patients with a low global LUS score, extubation can be expected in the first weeks of mechanical ventilation, whereas this is uncommon in patients with a high global LUS score. The extent of subpleural consolidations or pleural line abnormalities does not add prognostic value to the global LUS score in invasively ventilated patients.
